# Prescribing and sales of intramammary antimicrobials in Ireland in 2019 and 2020: the role of milk purchasers

**DOI:** 10.1186/s13620-022-00227-4

**Published:** 2022-11-19

**Authors:** Simon J. More, Jamie M. Madden, Catherine I. McAloon

**Affiliations:** 1 UCD Centre for Veterinary Epidemiology and Risk Analysis, University College Dublin, Belfield, Dublin, D04 W6F6 Ireland; 2 UCD School of Veterinary Medicine, University College Dublin, Dublin, Belfield D04 W6F6 Ireland

**Keywords:** Mastitis control, Intramammary antimicrobial stewardship, Veterinary prescribing, Ireland, Somatic cell count, Milk purchasers, Dairy industry

## Abstract

**Background:**

In Ireland between 2008 and 2022, intramammary antimicrobial (AM) products could be prescribed by a veterinary practitioner under what was known as Schedule 8 (or remote) prescribing. Under this prescribing route, an annual herd visit was not required when criteria were met as outlined in Animal Remedies Regulation 2007 to 2017 (statutory instruments No. 786/2007 and 558/2017). Under this prescribing route, the responsibilities of the milk purchaser, the farmer and the veterinary practitioner were each outlined, and a written mastitis control programme (MCP) was required. Milk purchasers implemented MCPs on participating farms (so-called MCP herds) with support from veterinary practitioner(s) who undertook Schedule 8 prescribing of intramammary AM tubes. This study seeks a clearer understanding of the role of milk purchasers in the prescribing and sale of intramammary AM products in Ireland during 2019 and 2020, whilst this Regulation was in force. Specifically, the study sought insights into the role of milk purchasers in the prescribing and sale of intramammary AM products in the Irish dairy industry during 2019 and 2020, using anonymised and highly aggregated milk purchaser data. The study also provided insights into milk quality among supplying herds during this period.

**Methods:**

For this study, we had access to anonymised, highly aggregated data from all milk purchasers that operated a MCP on at least some of their supplying herds during 2019 or 2020. Data collection was undertaken by the Department of Agriculture, Food and Marine. Data analysis was primarily descriptive.

**Results:**

Data were available on 11 milk purchasers (64.7% of all) and 13,251 supplying herds. Of these, 52% were MCP herds. The quality of milk from supplying herds varied significantly by month, year and milk purchaser. During 2019 and 2020, there was a single Schedule 8 prescriber (a private veterinary practitioner prescribing intramammary AMs as part of a MCP), on average, for 549.3 herds. The sale of intramammary AM products through milk purchasers represented 15.2% and 26.9% of national sales in in-lactation and dry cow tubes, respectively. There was an overall 2% increase in sales through milk purchasers between 2019 and 2020. Few European Medicines Agency (EMA) category B (‘*Restrict*’) intramammary AM products were sold by milk purchasers. For both in-lactation and dry cow tubes, there was a statistically significant association between EMA classification and route of sale (through milk purchasers or otherwise).

**Conclusions:**

The study findings provide important insights into mastitis control and intramammary AM stewardship in the Irish dairy industry. Significant differences between milk purchasers were observed in the quality of milk, as measured through somatic cell count (SCC) values, from supplying herds. This warrants further research. In the context of intramammary AM prescribing, veterinary oversight under the Animal Remedies Regulation 2007 to 2017 was very limited during 2019 and 2020. There were also significant associations between EMA classification and route of sale during 2019 and 2020, reinforcing the need for Irish veterinary practitioners to move away from EMA category B intramammary AMs. Higher quality data are needed to address important industry questions. Specifically it is recommended that national bulk tank SCC data are made available for public good research. Past experiences with Schedule 8 prescribing (no longer permitted from 28 January 2022) may influence current efforts towards improved intramammary AM stewardship.

**Supplementary Information:**

The online version contains supplementary material available at 10.1186/s13620-022-00227-4.

## Introduction

Within the Irish dairy sector, there is a strong cooperative history with substantial farmer ownership and control [1]. Currently there are 10 milk processors and 17 milk purchasers [1], and approximately 17,500 dairy farms and 1.2 million dairy cows, with an emphasis on seasonal, grass-based production [2]. Milk purchasers (cooperatives etc) play a role in sales of intramammary antimicrobial (AM) products to Irish dairy farmers and historically have also played a role in AM prescribing.

The Veterinary Council of Ireland establishes, publishes, maintains and reviews the professional code of conduct for veterinary practitioners in Ireland, as required under the Veterinary Practice Act 2005 [3]. In the version of the Code issued on 22 January 2018, it was stated that AMs may only be prescribed by a veterinary practitioner in the context of a client-patient-practice relationship (CPPR), which was defined as* ‘an agreement between an animal owner (or a keeper) and a veterinary practitioner(s) within a veterinary practice to provide veterinary services that demonstrate real and ongoing veterinary/animal contact rather than distant, nominal or bureaucratic veterinary input’ *[4]. It was further stated that the factors constituting prudent AM prescribing were considered to vary depending on the medicine, the species, the number of animals and environments in which they are treated, but that a maximum interval of 90 days between direct clinical examination of animal(s) and medicine prescribing could be expected to cover most practical situations. In the latest revision of the Code, issued on 09 December 2021 [5],the responsible use of medicinal products in animals incorporates the legislative requirements of Regulation (EU) 2019/6 (the Veterinary Medicines Regulation) [6], including the requirement for veterinary practitioners to prescribe an antimicrobial medicinal product only after a clinical examination or other proper assessment of the health status of the animal or groups of animals has been carried out, with justification required, in particular for metaphylaxis and prophylaxis.

For just over 14 years, from 01 January 2008 to 27 January 2022, there was an exemption to these broad principles with respect to the prescribing of intramammary AMs. Under the Animal Remedies Regulation 2007 to 2017 (statutory instrument (SI) No. 786/2007 [7] and 558/2017 [8]), intramammary AMs could be prescribed by a veterinary practitioner without the requirement for a herd visit at least every 12 months if the animal to be treated belongs to a herd covered by*‘a programme meeting the requirements of the Schedule’*, the objective of which was *‘the prevention and treatment of clinical and subclinical bovine mastitis in a manner designed to minimise use of antibiotic treatments and, where necessary, set targets for a reduction in the number of mastitis cases for that herd’*. Under this prescribing route, which has variously been termed ‘Schedule 8 prescribing’ or ‘remote prescribing’, the responsibilities of the milk purchaser, the farmer and the veterinary practitioner were each outlined, and a written mastitis control programme (MCP) was required. As specified in this legislation, there was a requirement for the milk purchaser to implement a structured sampling programme (including AM residues, somatic cell counts (SCCs), pathogen isolation and AM susceptibility testing where appropriate, and total bacterial count), and for the farmer to maintain a record of the number of cows in the herd during lactation, the number of cows infected with mastitis, the number of cows treated and the number of intramammary animal remedies used. The registered veterinary practitioner (the remote prescriber) was required to take full consideration of data and of the general health situation on the farm, including the milking operation, and to review programme effectiveness. In summary, milk purchasers implemented MCPs on participating farms (so-called MCP herds) with support from veterinary practitioner(s) who undertook Schedule 8 prescribing of intramammary AM tubes.

SI No. 786/2007 was revoked (and Schedule 8 prescribing ceased) with the introduction on 28 January 2022 of the European Union (Veterinary Medicinal Products and Medicated Feed) Regulations 2022 (SI No. 36/2022) [9]. Although SI No. 558/2017 [8] remains on the Statute Books, Article 105(3) in Regulation (EU) 2019/6 [6] and the Veterinary Council of Ireland (VCI) Code of Professional Conduct for Veterinary Practitioners (2021 revision) [5] working in tandem only permit a veterinary practitioner to prescribe within a CPPR, which the previous MCPs do not fulfil.

In Ireland, milk purchasers continue to have a role in the sales of intramammary AM products, noting that these may only be supplied on prescription. This role has not changed with the introduction of the Veterinary Medicines Regulation [6]. Prescribed AMs can be dispensed by a veterinarian or pharmacist, in accordance with the prescription of the prescribing veterinary practitioner. In addition, intramammary AM products can be dispensed by licensed agricultural merchants, including milk purchasers.

The current study seeks a clearer understanding of the role of milk purchasers in the prescribing and sale of intramammary AM products in Ireland during 2019 and 2020, whilst the Animal Remedies Regulation 2007 to 2017 [7, 8] was in force. There were two related drivers in support of the current study. Firstly, the study will fill a key knowledge gap for scientists and policy makers. For some years, scientific research has been conducted, including by this research group, to better understand the constraints to, and opportunities for, improved national mastitis control and AM stewardship in the Irish dairy industry. To this point, limited national data have been available in support of AM stewardship in the dairy industry. Insights are available on milk quality in the national herd [10, 11], noting the close linkage between mastitis control and AM usage. In the absence of a national prescribing database, trends in AM usage have relied on national AM sales data, including an understanding of trends in sales during 2003 to the present [10, 12–14]. As yet, limited published information is available on the role of milk purchasers, either in Schedule 8 prescribing or on AM sales. Secondly, the study is motivated by questions from the dairy industry. In particular, the industry has been seeking an understanding of the long-term impact of the Animal Remedies Regulation 2007 to 2017 [7, 8] on milk quality and AM stewardship in MCP herds, and of the contribution of milk purchasers to national patterns of intramammary AM sales.

This study forms part of broader work in support of CellCheck, the national mastitis control programme, which is coordinated by Animal Health Ireland [15]. The study will inform technical discussions (including in the CellCheck Technical Working Group (TWG) [16]) and policy decision-making by government and industry, the latter within the CellCheck implementation group (IG) [17]. This work is being conducted in support of two action points in Ireland’s second One Health National Action Plan on Antimicrobial Resistance (2021–2025) (iNAP2) [18], relevant to the prudent use of AMs in the Irish dairy industry, including the development and implementation of ‘a system for the collection of data in relation to usage of intramammary tubes in the dairy sector’ (2.21, under *Strategic objective 2: enhance surveillance of antibiotic resistance and antibiotic use)* and implementation of ‘measures to improve the national Somatic Cell Count through the CellCheck programme’, which is Ireland’s national programme to facilitate improved mastitis control in the dairy industry (3.25, under *Strategic objective 3: reduce the spread of infection and disease).*

Therefore, the aim of this study was to provide insights into the role of milk purchasers in the prescribing and sale of intramammary AM products in the Irish dairy industry during 2019 and 2020, using anonymised and highly aggregated milk purchaser data. The study also provided insights into milk quality among supplying herds during this period.

## Materials and methods

Under the Animal Remedies Regulation 2007 to 2017 [7, 8], the Department of Agriculture, Food and Marine (DAFM) is responsible for collecting data from all milk purchasers in Ireland that operated a MCP on at least some of their supplying herds.

A data collection template was developed to assist with data collection, noting that data were sought from each milk purchaser, separately for 2019 and 2020. Grouped by MCP participation (either all supplying herds (MCP and non-MCP herds) or MCP herds only) and unit of interest, these data included:

In all supplying herds:

- *Herd.* The aggregate total number of herds (noting that data were not available at herd level).
- *Herd-month.*During each month, counts of the total number of herds supplying milk to the milk purchaser and of the number of herds by monthly herd SCC value of milk. Consistent with Trader Notice DH/TN/01/2018 (revised May 2018) [19] from DAFM, the monthly SCC value for each herd was calculated as the geometric mean of all SCC values measured during that month. The monthly herd SCC value was available as a categorical variable, either < 200,000, 200,000–299,000, 300,000–399,000, 400,000–749,000, 750,000–999,000 or ≥ 1,000,000) cells/mL. All herd-months in the latter three categories (ie > 400,000 cells/mL) were counted as SCC exceedances.
- *Intramammary AM tube.* The total number of intramammary AM tubes sold, by treatment (in-lactation, dry cow) and product name.

In MCP herds only:

- *Herd.* The aggregate total number of herds implementing a MCP (so-called MCP herds).
- *SCC and AM test.* The number of herd-level SCC and AM residue tests conducted each month.
- *SCC and AM breach.*The number of breaches notified each month. As outlined in DAFM Trader Notice DH/TN/01/2018 (revised May 2018) [19], a SCC breach is defined as a geometric mean SCC value exceeding 400,000 cells/mL, based on all sample results over the previous three-month period, with at least one sample per month. An AM breach refers to the detection of a prohibited AM in a tested milk sample.
- *People.* The number of veterinary practitioners providing professional oversight of the MCP, the number of other veterinary practitioners assigned a formal role under the MCP, the number of other persons assigned a formal role under the MCP.
- *Prescriptions.* The total number of prescriptions issued, the number of prescriptions issued for in-lactation and for dry cow therapy.
- *Intramammary AM tube.* The total number of AM tubes prescribed for in-lactation and for dry cow therapy, by treatment (in-lactation, dry cow) and product name.

The data collection template was issued by DAFM to each of these milk purchasers. The completed data sheets were returned to DAFM, then (following anonymisation of the identify of each milk purchaser) forwarded to the authors. The milk purchasers were identified as A through to K. The authors conducted a detailed review of these data and documented a range of questions relating to potential errors or data quality issues. These questions were sent back to each milk purchaser via DAFM. The data sheets were subsequently revised by the milk purchasers. The revised anonymised data sheets formed the basis of the current analysis.

Descriptive analytical methods were primarily used, with data presented as mean, median or interquartile range where appropriate. The supply of milk from herds to milk purchasers was assumed to only occur in those calendar months when SCC testing was recorded. The average annual duration of supply was calculated by dividing the total number of herd-months by the total number of herds, in total and by milk purchaser. AMs were classified with regard to their risk to public health using the classification system outlined by the European Medicines Agency (EMA) [20]. We used national sales from McAloon et al. [13, 14] when calculating the percentage of national intramammary AM tubes that were sold by milk purchasers during 2019 and 2020. SCC exceedance was considered to occur when the monthly herd SCC value was 400,000 cells/mL or greater.

The association between categorical variables (for example, EMA classification and route of sale) were assessed using the chi square test. A value of *P* < 0.05 was considered statistically significant. The association between milk purchaser and SCC exceedance (> 400,000 cells/mL) was assessed using a logistic regression with adjustment for year and month. Unfortunately, herd ID was not included during data collection. Therefore, although we had correlated data (repeated measurements from some herds), we did not know which data formed a repeated measurement. In an ideal situation, herd ID (if available) would have been incorporated as a random-effect to account for this correlation. As a crude way to overcome this, we calculated confidence intervals (CIs) and the odds ratio (OR) using robust standard errors.

Data management and analyses were undertaken using Microsoft Excel and R software (R Core Team, 2021).

## Results

Available data were aggregated, and were not available at herd-level.

### All supplying herds

#### Number of herds

In total, data were available from 11 milk purchasers, each operating a MCP in the years of interest. That is, 64.7% of all 17 Irish milk purchasers. These 11 milk purchasers had a total of 13,284 and 13,217 supplying herds during 2019 and 2020, respectively, with considerable variation between milk purchasers (median of 1,205 supplying herds per milk purchaser per year during 2019 and 2020, interquartile range 219–1,819).

#### Duration of supply

**Fig. 1:**
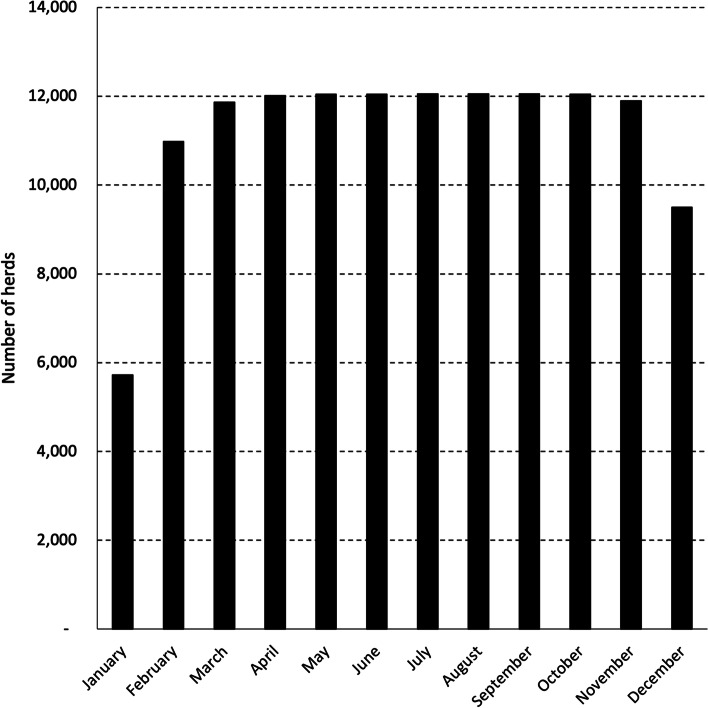
The number of herds supplying milk to the 11 milk purchasers each month, during 2019 and 2020

During 2019 and 2020, the overall average annual duration of supply was 10.1 months (among purchasers, the median annual duration of supply was 10.8 months; interquartile range 10.7–11.2). Supply was seasonal, being lowest in January (43% of all herds supplying) and December (72% of all herds supplying) (Fig. 1).

#### Milk quality

**Fig. 2:**
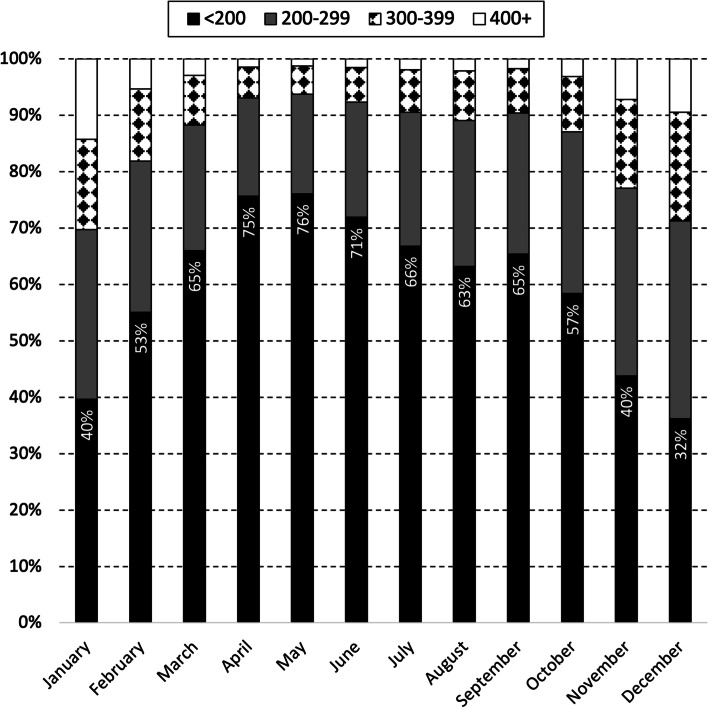
Distribution of monthly herd SCC values among supplying herds during 2019 and 2020, by calendar month. As outlined in Trader Notice DH/TN/01/2018 (revised May 2018) from DAFM to milk purchasers, the monthly herd SCC value for each supplying herd is the geometric mean based on all available SCC values for that month. The percentage of supplying herds with monthly mean SCC values below 200,000 cells/mL is indicated. To illustrate, 40% of the monthly SCC values from herds supplying to milk purchasers during January 2019 and January 2020 was below 200,000 cells/mL

There is a pronounced seasonal pattern in milk quality, as measured using SCC categories, from supplying herds during 2019 and 2020 (Fig. 2). The distribution of monthly herd SCC values by calendar month, but separately for 2019 and 2020, is presented in Figures S1 and S2 in the supplementary material. Milk quality is highest mid-year (in May, 76% of supplying herds had a monthly herd SCC value < 200,000 cells/mL) and substantially lower at the start and end of each year (40% of supplying herds in January, 32% in December) (Fig. 2).

**Fig. 3:**
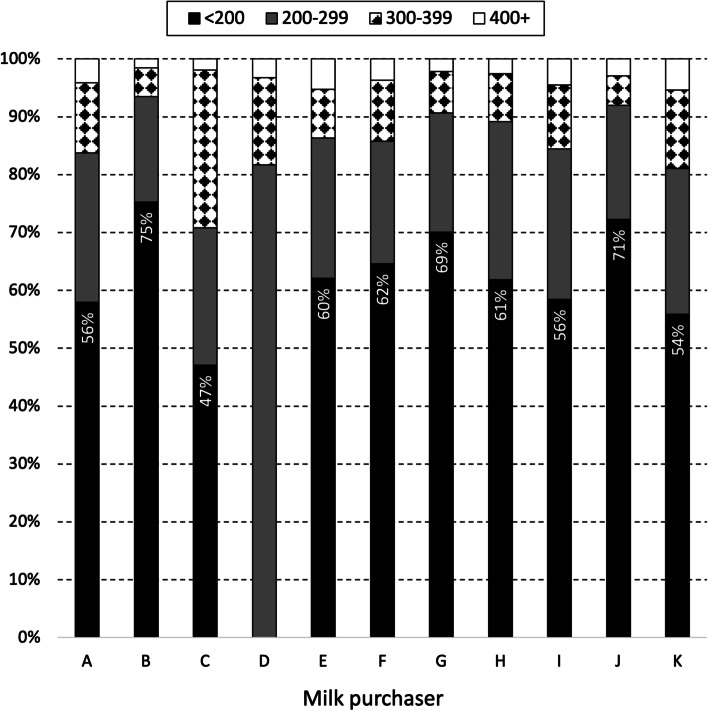
Distribution of monthly herd SCC values among supplying herds during 2019 and 2020, by milk purchaser. Using methods as outlined in Trader Notice DH/TN/01/2018 (revised May 2018) from DAFM to milk purchasers, the monthly herd SCC value for each supplying herd is the geometric mean based on all available SCC values for that month. The percentage of supplying herds with monthly SCC values below 200,000 cells/mL is indicated. To illustrate, 56% of the monthly SCC values from herds supplying to milk purchaser A during 2019 and 2020 was below 200,000 cells/mL

The quality of milk from supplying herds during 2019 and 2020 varied significantly between milk purchasers (Fig. 3, X^2^ = 6,613.6, *p* < 0.001). Milk quality was highest with milk purchaser B; 75% of the monthly herd SCC values from supplying herds during 2019 and 2020 was < 200,000 cells/mL. The distribution of monthly herd SCC values among supplying herds by milk purchaser, but separately for 2019 and 2020, is presented in Figures S3 and S4 in the supplementary material.

Output from the logistic model exploring the association between purchaser and the occurrence of an exceedance (monthly herd SCC > 400,000 cells/mL) is presented in Table 1. In comparison to purchaser B, purchaser I was statistically significantly associated with an exceedance and had the largest odds ratio (OR = 5.09, CI: 4.16 – 6.22) after adjustment for year and month. A one unit increase in year (from 2019 to 2020) was associated with a reduction in the chance of an SCC exceedance (OR = 0.87, CI: 0.84 – 0.90) which reflects the drop in the total number of exceedances from 2019 to 2020 (8,797 versus 8,005). In comparison to April, December and January had the highest odds ratios (OR = 10.72 (CI: 9.80 – 11.74) and 7.58 (6.87 – 8.36) respectively).

**Table 1:** Association between several independent variables (calendar month, year and milk purchaser) and SCC exceedance (> 400,000 cells/mL) using logistic regression, among supplying herds during 2019 and 2020

Variable	Odds ratio	95% confidence interval
Month		
January	7.58	6.87—8.36
February	4.42	4.02—4.85
March	2.18	1.97—2.41
May	0.75	0.66—0.85
June	0.94	0.83—1.06
July	1.10	0.98—1.24
August	1.25	1.11—1.39
September	1.06	0.94—1.19
October	2.11	1.91—2.34
November	7.02	6.41—7.68
December	10.72	9.80—11.74
Year (2020)	0.87	0.84—0.90
Milk purchaser		
A	4.02	3.27—4.94
C	1.03	0.71—1.49
D	3.49	2.52—4.83
E	4.65	3.75—5.77
F	3.51	2.87—4.30
G	1.92	1.54—2.39
H	2.13	1.74—2.61
I	5.09	4.16—6.22
J	2.30	1.82—2.91
K	4.21	3.40—5.22

The referent categories include milk purchaser B, 2019 and April

### MCP herds only

#### Number of herds

There were 6,729 and 7,004 MCP herds in 2019 and 2020, respectively, being an average of 52% of supplying herds to these 11 milk purchasers. Among milk purchasers, there was variation in the percentage of all supplying herds that were MCP herds (median 62%, interquartile range 43–78%).

#### SCC and AM testing and compliance

During 2019 and 2020, an average of 148.7 SCC tests/herd and 27.0 AM tests/herd were conducted annually, but this varied between milk purchasers (for SCC tests: median 112.0 tests/herd/year, interquartile range 94.1–136.3; for AM tests: median 19.3 tests/herd/year, interquartile range 1.7–97.2).

SCC breaches were relatively common, whereas AM breaches are less so, however, this varied between milk purchasers. During 2019 and 2020, there were 64.2 SCC breaches per 100 herds per year (among milk purchasers: median 62.9, interquartile range 10.7–86.0), primarily in November and December. This is equivalent to, on average, 64.2% of farms experiencing a single SCC breach per year (among milk purchasers: median 62.9%, interquartile range 10.7–86.0%). Similarly, there were 4.4 AM breaches per 100 herds per year (among milk purchasers: median 2.4, interquartile range 1.2–4.3), most commonly in February, November and December. This is equivalent to, on average, 4.4% of farms experiencing a single AM breach per year (among milk purchasers: median 2.4%, interquartile range 1.2–4.3%).

**Fig. 4:**
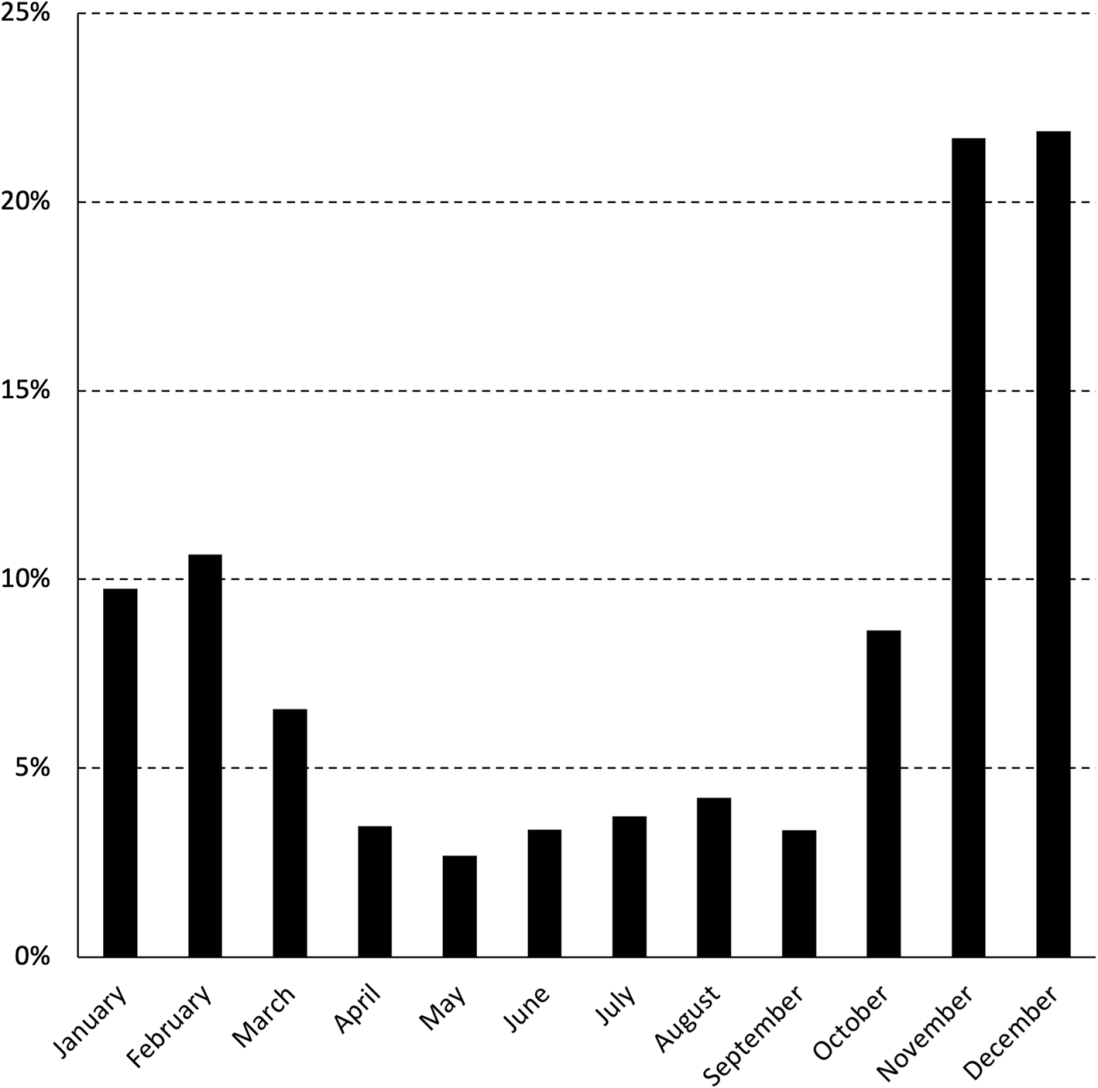
Frequency of SCC breaches in MCP herds during 2019 and 2020, by calendar month. Of the 8,817 SCC breaches reported in MCP herds during 2019 and 2020, 860 (9.8%) occurred in January

**Fig. 5:**
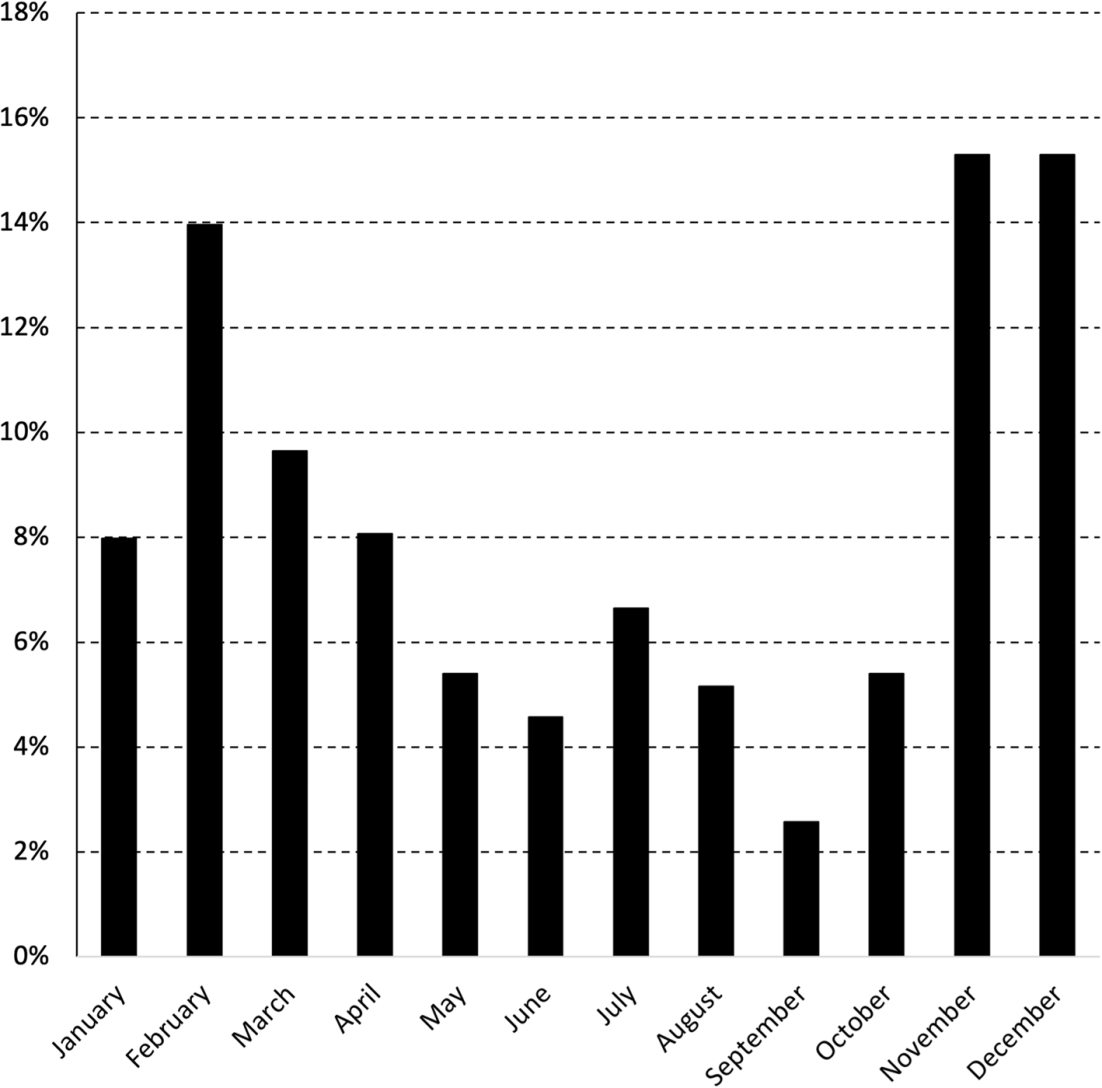
Frequency of AM breaches in MCP herds during 2019 and 2020, by calendar month. Of the 602 AM breaches reported in MCP herds during 2019 and 2020, 48 (8.0%) occurred in January

The frequency of SCC and AM breaches in MCP herds during 2019 and 2020, by calendar month, is presented in Figs. 4 and 5. To illustrate, of the 8,817 SCC breaches reported in MCP herds during 2019 and 2020, 860 (9.8%) occurred in January (Fig. 4). No data were collected on the number of MCP herds supplying milk to the 11 milk purchasers each month, but this is likely to be similar to the pattern observed for the total number of supplying herds (Fig. 1).

#### Veterinary oversight

Veterinary oversight of MCP herds by veterinary practitioners operating within Schedule 8 was very limited. During 2019 and 2020, a single veterinary practitioner had oversight, on average, of 549.3 MCP herds. However, there was considerable variation between milk purchasers (median 230.5 herds per veterinary practitioner, interquartile range 122.3–510.9), and at its most extreme, a single veterinary practitioner had oversight of 2,443 herds in 2019 and 2,398 herds in 2020. Under each MCP, other people are also assigned a formal role. With the inclusion of all assigned people (veterinary practitioner and other people), oversight increased to an average of 1 assigned person to 144.5 herds. Again, there was considerable variation between milk purchasers (median 77.8 herds per assigned person, interquartile range 43.7–163.5).

#### Intramammary AM prescribing

In most cases during 2019 and 2020, a single prescription (mean 1.02) was written for each MCP herd each year. However, there was some variation between milk purchasers (median 1.0 prescription per MCP herd per year, interquartile range 0.95–1.05). There was an average of 158.6 in-lactation tubes prescribed per herd per year (among milk purchasers: median 193.3 in-lactation tubes prescribed per MCP herd per year, interquartile range 96.3–265.5), and an average of 527.1 dry cow tubes prescribed per herd per year (among milk purchasers: median 428.7 dry cow tubes prescribed per herd per year, interquartile range 226.7–749.0).

### Sales of intramammary AM tubes to all supplying herds

#### Overall sales

During 2019 and 2020, the 11 milk purchasers sold 2,953,421 intramammary AM tubes to all supplying herds (both MCP and non-MCP), including 564,472 in-lactation and 2,388,949 dry cow tubes, representing an estimated 15.2% and 26.9% of national sales in in-lactation and dry cow tubes, respectively. Compared with 2019, the 2020 sales represented a 4.3% reduction in the sales of in-lactation tubes, a 3.5% increase in the sales of dry cow tubes, and a 2% increase overall.

#### In-lactation tubes sales

**Fig. 6:**
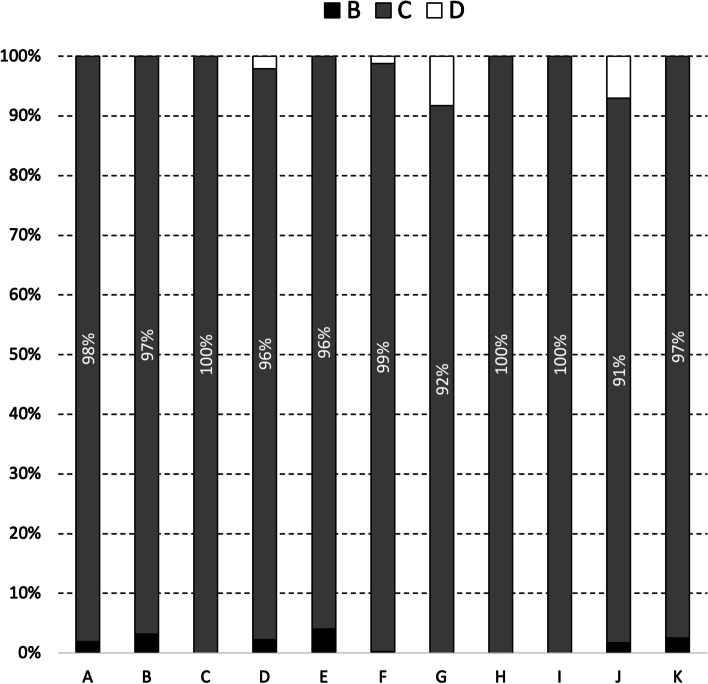
Distribution of EMA classification (B [*'Restrict'*], C [*'Caution'*] or D [*'Prudence'*]) of in-lactation tubes sold by different milk purchasers during 2019 and 2020. The percentage of in-lactation tubes with an EMA classification of C is indicated

With respect to in-lactation therapy during the study period, a mean of 21.3 tubes were sold per supplying herd per year, varying between milk purchasers (median 28.2 in-lactation tubes/supplying herd/year per milk purchaser; interquartile range 14.3–54.5). Of the tubes sold, 3,734 (0.7%) were EMA classification B *(‘Restrict’*), 554,706 (98.2%) were classification C *(‘Caution’*) and 6,032 (1.1%) were classification D *(‘Prudence’*). These relative proportions in EMA classification were similar across milk purchasers (Fig. 6). Milk purchaser G did not sell any classification B tubes and sold more classification D tubes compared to other purchasers as a total of each purchaser’s sales. The distribution of EMA classification of in-lactation tubes sold by different milk purchasers, but separately for 2019 and 2020, is presented in Figures S5 and S6, respectively, in the supplementary material.

There is a significant association between EMA classification and route of sale (sales from milk purchasers, national sales apart from milk purchasers) for in-lactation tubes sold during 2019 and 2020 (X^2^ = 65,778, *p* < 0.001).

#### Dry cow tube sales

**Fig. 7:**
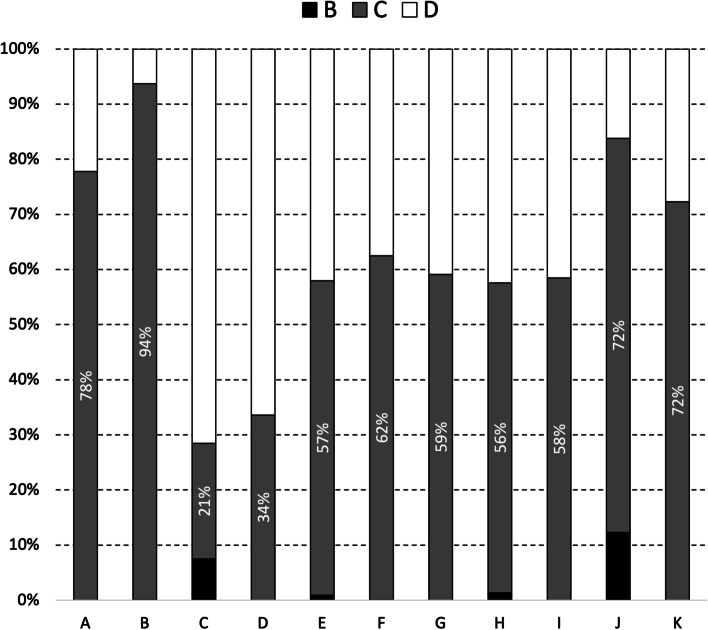
Distribution of EMA classification (B [*'Restrict'*], C [*'Caution'*] or D [*'Prudence'*]) of dry cow tubes sold by different milk purchasers during 2019 and 2020. The percentage of dry cow tubes with an EMA classification of C is indicated

With respect to dry cow therapy during this period, an average of 90.1 tubes were sold per supplying herd per year, varying between milk purchasers (median 84.9 dry cow tubes/supplying herd/year; interquartile range 53.4–157.4). Of the tubes sold, 21,798 (0.9%) were EMA classification B *(‘Restrict’*), 1,417,638 (59.5%) were classification C *(‘Caution’*) and 949,513 (39.7%) were classification D *(‘Prudence’*). These relative proportions in EMA classification varied between milk purchasers (Fig. 7). The distribution of EMA classification of dry cow tubes sold by different milk purchasers, but separately for 2019 and 2020, is presented in Figures S7 and S8, respectively, in the supplementary material.

There is a statistically significant association between EMA classification and route of sale (sales from milk purchasers, national sales apart from milk purchasers) for dry cow tubes during 2019 and 2020 (X^2^ = 331,931, *p* < 0.001). Milk purchasers J and C sold the highest percentage of classification B AMs during this period, comprising 12.3% and 7.5%, respectively.

#### Comparison of prescribing and sales of intramammary AM tubes

In general, the number of tubes prescribed to MCP herds was substantially greater than the number of tubes sold by milk purchasers to all supplying herds. For in-lactation tubes, on average there were 1.93 more tubes prescribed than sold (among milk purchasers: median 1.64 in-lactation tubes prescribed/tubes sold, interquartile range 1.0–2.1), and for dry cow tubes an average of 1.52 more tubes prescribed than sold (among milk purchasers: median 1.64 dry cow tubes prescribed/tubes sold, interquartile range 1.0–1.5).

## Discussion

### The context

This study was conducted with the aim to provide insights into the role of milk purchasers in the prescribing and sale of intramammary AM products in the Irish dairy industry during 2019 and 2020. The study also provided insights into milk quality among supplying herds during this period. This information will contribute to ongoing efforts, including by this research group, to better understand the constraints to and opportunities for improved national mastitis control and AM stewardship in the Irish dairy industry. This information helps to inform technical discussions, including in the CellCheck TWG, and policy decision-making, by government and also at an industry level by the CellCheck IG. This background information is particularly important in the context of Veterinary Medicines Regulation [6], which places particular emphasis on AM stewardship [21–23].

With the introduction of SI No. 36/2022 [9], aspects of this study relating to Schedule 8 prescribing will relate to past, rather than current, practices. However, there are a few reasons why these study results are of current relevance. Firstly, this work is motivated by questions posed both by government (DAFM Veterinary Medicines Division) and by the CellCheck IG over a series of years. Progress prior to 2021 was constrained by difficulties relating to data quality and availability. These challenges have been substantially addressed following a heightened role by DAFM during data collection. Secondly, Schedule 8 prescribing has been an important feature of the prescribing landscape in Ireland from 2007 to 2022. AM stewardship will likely be a contended space in the future, and it is important that Schedule 8 prescribing is objectively documented, both to ensure that it is clearly understood in the future and also as a means to highlight lessons learned. Thirdly, past practices will influence current and future thinking with respect to AM stewardship in the Irish dairy industry. We need an accurate understanding of these practices if we are to successfully shape change into the future. Fourthly, the work is part of a broader series of studies investigating constraints to and opportunities for improved mastitis control and intramammary AM usage in the Irish dairy industry [10–14, 21–26]. Schedule 8 prescribing has been an important influencer of both of these issues, and cannot be ignored in this broader discussion. Finally, these data provide insights into milk quality and AM stewardship in a large subset of Irish dairy herds. To this point, we do not have access to the national bulk tank SCC data for public good research, and are reliant on other data sources to assist us with our work, including this current, highly summarised dataset from milk purchasers.

### Milk quality among supplying herds

These data highlight the substantial improvement to national milk quality that has occurred between 2003–10 (percentage of herds exceeding 400,000 cells/mL varied between 9% in May 2007 and 45% in December 2009 [10]) and 2019–20 (1% in April–May 2019 and April-June 2020 and 15% in January 2019). This is in agreement with recent published information [23]. The CellCheck mastitis control programme, which commenced in 2011 [11], is an important national initiative, which is accepted to have at least partly contributed to this improvement, although no objective assessment has been undertaken.

In 2019 and 2020, significant differences in the quality of milk from supplying herds were observed by month, year and milk purchaser (Table 1). The seasonal pattern of milk quality in Ireland is readily apparent, being highest mid-year and lowest at the start and end of each year (Table 1, Fig. 2). This observation is consistent with earlier findings [10], despite key differences between the two studies. The work by More et al. [10] considered the period 2003–2010 (compared with 2019 and 2020 here), herd-level data at each milk recording (compared to herd-level data based on a geometric mean monthly value here), between 3,296 and 6,400 milk recording herds (depending on the year) (compared with 13,284 herds in 2019 or 13,217 herds in 2020) supplying to 11 milk purchasers). Milk production is highly seasonal in Ireland and it has been suggested that the observed pattern could reflects a dilution effect during peak production. However, this hypothesis was not supported by Boland et al. [27], who found no evidence of a dilution effect in Irish dairy cattle. The increase in SCC breaches at the beginning of the year (Fig. 4) may be linked with an increasing number of herds that milk over the winter period. Conversely, AM breaches at the end of the year, as highlighted in Fig. 5, are likely related to management errors around the drying off process. Compared with 2019, there was a significant reduction in the chance of an SCC exceedance in 2020 (Table 1). Reasons for this are uncertain, however, it has been speculated that year-on-year variation in weather, among other factors, is a possible consideration. The significant difference in milk quality between milk purchasers is an important finding (Table 1, Fig. 3), and has not been previously reported. At its most extreme, milk purchaser I was five times more likely to have supplying herds with SCC greater than 400,000 cells/mL compared with supplying herds to milk purchaser B (Table 1). Further investigation is needed, noting that an understanding of the reasons for these differences may offer opportunities for national improvement. Consistent with this finding, the number of SCC breaches per 100 herds per year (or the percentage of farms experiencing, on average, a single SCC breach per year) also varied between milk purchasers, with an interquartile range of 10.7–86.0 (10.7–86.0%). In Ireland, an SCC breach cannot occur during the first three months following a break-in-supply, due to the way that EU legislation is interpreted [25]. The seasonal pattern in notification of SCC breaches (Fig. 4) is consistent with this, being highest in the latter part of the year.

### AM stewardship/veterinary oversight of AM prescribing

The concept of AM stewardship is central to international, European and national efforts to limit AM resistance (AMR), with AM stewardship referring to efforts made to ensure that AMs are used only when necessary and appropriate. In the global action plan on AMR, adopted by the World Health Organization (WHO) in 2015, with support from the Food and Agriculture Organization of the United Nations (FAO) and the World Organisation for Animal Health (WOAH, founded as OIE), responsible and prudent use of these medicines in human and animal health is a key goal. In food animal production, AM stewardship refers to efforts to limit inappropriate usage, and to optimise the choice, dose rate, route and duration of therapy to maximise clinical cures [28, 29]. Veterinary oversight of AM prescribing and use is central to these efforts. At the European level, prudent AM usage and veterinary oversight are critical to the Veterinary Medicines Regulation [6], and within Ireland, best-practice underpinning veterinary oversight of AM prescribing is outlined in the recently revised national Code of Professional Conduct for Veterinary Practitioners [5].

Concerns about prescribing under the Animal Remedies Regulation 2007 to 2017 [7, 8] have been raised on a number of occasions, mainly in the context of veterinary supervision and oversight. For example, the Food Safety Authority of Ireland do not support differential oversight of intramammary AMs in food animal production, and recommended that prescribing controls be changed ‘*to ensure that the level of veterinary supervision required in relation to use of antimicrobial agents in intramammary formulations is equivalent to the level that applies in most other prescribing scenarios*’ [30]. Further, McAloon et al. [13] suggest that*‘this prescribing route is unlikely to provide the veterinary oversight necessary to support prudent prescription decision making on the basis of a detailed, on-farm understanding of mastitis and farm management’* and *‘recommend an urgent review of overall prescribing practices for intramammary antimicrobials in the context of responsible AM stewardship’*.

The current study confirms these concerns. In the context of intramammary AM prescribing, veterinary oversight under the Animal Remedies Regulation 2007 to 2017 [7, 8] was very limited during 2019 and 2020, with a single veterinary practitioner providing oversight of Schedule 8 prescribing on an average of 549.3 MCP herds. Although there was considerable variation between milk purchasers, at its most extreme a single veterinary practitioner prescribed intramammary AMs to 2,241 MCP herds. These prescribers under the Animal Remedies Regulation 2007 to 2017 [7, 8] will likely have little or no knowledge of other AMs (including potentially other intramammary AMs) that are entering the farm, including by the attending (i.e. local) veterinary practitioner. In the absence of any requirement for a single prescribing veterinary practitioner or veterinary practice per farm, AMs could also be sourced from more than one veterinary practitioner, with prescribing decisions being made without knowledge of any other AM sources. This conclusion is relevant both to MCP and non-MCP herds.

These findings have implications both for veterinary oversight of AM prescribing but also for professional oversight of optimal mastitis control. These concepts are linked, noting the challenges faced with AM stewardship in those one-third of Irish herds where the annual geometric mean bulk tank SCC exceeding 200,000 cells/mL [23], suggestive of suboptimal mastitis control (the equivalent figure for MCP herds is unknown, as national bulk tank SCC data are not currently available). In these higher SCC herds, mastitis control is more challenging, given the greater proportion of infected cows. Lower levels of hygiene and general farm management are commonly associated with both environmental and contagious mastitis. Further, with contagious mastitis, ongoing infection pressure will facilitate the spread of infection, particularly during milking [23]. As outlined elsewhere [22, 23], sustainable improvement to mastitis control on these farms would require a detailed farm investigation by the veterinary practitioner, in partnership with the farmer and other milk quality professionals, to understand the epidemiology and on-farm drivers of mastitis, to develop farm-specific action plans, and to facilitate ongoing monitoring of progress. Based on information from the current study, the observed level of veterinary oversight by veterinary practitioners under the Animal Remedies Regulation 2007 to 2017 [7, 8] on these MCP herds appears to provide no realistic opportunity for any meaningful contribution to resolving sub-optimal mastitis control.

It is important to reflect on levels of veterinary oversight conducive to AM stewardship. Several studies have considered this issue in some detail. In the literature, factors critical to successful implementation of AM stewardship on dairy farms included those relating to the veterinary practitioner (concern for the role of veterinary AM use in development of AMR in humans, a sense of pride in the service provided, preparedness to change prescribing practices), the veterinary practitioner and farmer client (the strength of relationship between the veterinary practitioner and their farmer clients, the need for buy-in by dairy producers and employees, investing in the prevention of disease during critical moments of the production cycle, targeting undifferentiated use of AMs) and supporting tools (means to track on-farm AM usage, standardised treatment protocols, the availability of laboratory data) [31–33]. As perceived internationally by veterinary practitioners, barriers to successful implementation of AM stewardship included a lack of antimicrobial stewardship governance structures (including uncertainty about regulations for monitoring on-farm AM use,) variable relationships with clients and farm employees, client expectations and competition between practices, a lack of economic data in support of AM stewardship, cost of microbiological testing, and lack of access to education, training and AM stewardship resources [32, 33]. Further, it was suggested that training would assist veterinary practitioners to prescribe prudently in the face of potentially inappropriate farmer pressure [34]. Examples from Denmark and the Netherlands provide examples of international best-practice in AM stewardship in food animal production, noting that veterinary oversight is just one element within a multi-faceted national approach [23]. In Denmark, veterinary advisory service contracts have been mandated in larger herds since 2010, requiring frequent veterinary visits and a 1-to-1 relationship between the farmer and the veterinarian. Farmer access to AMs is linked to the level of farm oversight that is provided by the veterinarian. Treatment and control measures are underpinned by an understanding of the aetiologic agent (i.e. bacterial culture), patterns of udder infections and AMR in each herd, and the use of narrow-spectrum AMs and selective DCT has become the norm [35]. A requirement for Dutch farmers to procure veterinary services and veterinary medicines from a single veterinary practice was introduced in 2009, in part to ensure a sound understanding of the farm by the prescribing veterinary practitioner [36].

### AM sales

The study results provide an estimate of the market share of national sales by the milk purchasers during 2019 and 2020, including 15.2% of in-lactation tubes and 26.9% of dry cow tubes. This information, which was not previously available, highlights the importance of these sales routes within Ireland. As yet, we are not aware of similar published data from other countries on sales by route of supply to allow comparison. For both regulatory and commercial reasons, routes of sale are likely to vary between EU member states.

A number of additional results relating to AM sales from milk purchasers are presented, including the EMA classification of intramammary AM tubes (see below), the association between EMA classification and route of supply, and a comparison between Schedule 8 prescribing and AM sales from milk purchasers. However, interpretation of these results, which themselves are based on summarised data, will only be straightforward if AM sales from milk purchasers can be directly linked to Schedule 8 prescribing, and AM sales from other sources to non-Schedule 8 prescribing. However, this may not be the case, for several reasons. Firstly, there is no restriction in Ireland on the number of AM prescribers per herd. Therefore, MCP herds could potentially access intramammary AMs outside the formal MCP. Secondly, farmers are able to purchase AMs from multiple sources, provided each is covered by a prescription from a veterinary practitioner. MCP herds could purchase some or all of their prescribed intramammary tubes from a source other than their milk purchaser. Similarly, AM sales from milk purchasers could feasibly include both MCP and non-MCP herds.

Significant associations were identified between EMA classification and route of sale (both for in-lactation and dry cow AM tubes). We caution that these results need to be interpreted with care, for the reasons given above. Nonetheless, this result is in general agreement with an earlier finding by More et al. [12], who found that critically important AMs (CIAs) and highest priority CIAs (HP CIAs) intramammary products (these terms being used by the WHO [37], but are roughly equivalent to EMA classifications B and C [20]) were less likely to be prescribed through Schedule 8 prescribing compared with the routine prescribing route, the exception being the use of CIAs for in-lactation therapy. If correct, this result is of concern, with respect to prescribing decisions by veterinary practitioners to non-MCP herds. We note that consistent messaging in support of change was being introduced during the current study period (2019–20), including the EMA guidelines in 2019 [20] and detailed guidelines for Irish veterinary practitioners in 2020, both from government [38] and Animal Health Ireland [39]. Further, a shift in prescribing patterns has occurred more quickly with Schedule 8 prescribing, reflecting the ability (given the small number of prescribers involved) for the dairy industry to rapidly pivot from the use of category B intramammary AMs. As one example, during this period a decision was made by at least some milk purchasers to not stock intramammary AMs containing HP CIAs. Ongoing monitoring of this issue will be important, including through national AM sales data, to ensure a similar shift in prescribing by all veterinary practitioners is also observed.

### Conclusions

This study provides insights into the role of milk purchasers in the prescribing and sale of intramammary AM products in the Irish dairy industry during 2019 and 2020. The study also provided insights into milk quality among supplying herds during this period. There are a number of important study findings. Significant differences between milk purchasers were observed in the quality of milk, as measured through SCC values, from supplying herds. In the context of intramammary AM prescribing, veterinary oversight under the Animal Remedies Regulation 2007 to 2017 [7, 8] was very limited during 2019 and 2020, with a single Schedule 8 prescriber (a private veterinary practitioner prescribing intramammary AMs as part of a MCP), on average, for 549.3 herds. Although this latter finding is now of historic interest, given that Schedule 8 prescribing is no longer permitted under SI No. 36/2022 [9], these past experiences may influence ongoing efforts towards improved intramammary AM stewardship. There were also significant associations between EMA classification and route of sale during 2019 and 2020, reinforcing the need for Irish veterinary practitioners to move away from EMA category B intramammary AMs.

With many of these findings, further investigation is warranted. Unfortunately, this is not possible using the highly summarised data available to us. Similarly, it was not possible with the current data to evaluate the impact of Schedule 8 prescribing (and the broader MCP, as reflected in the Animal Remedies Regulation 2007 to 2017 [7, 8]) on milk quality and AM stewardship in MCP herds, these being key questions that motivated this study. In the current study, each of the milk purchasers provided highly aggregated data, using a purpose-built template guided by national legislation [7, 8] and the DAFM Milk Circular (Trader Notice DH/TN/01/2018, revised May 2018 [19]). In order to answer these, and a number of other, important industry questions, it is recommended that the national bulk tank SCC data are made available for public good research. DAFM have outlined the legal basis for sharing these data for this purpose [40]. Further, it will be important that these data include a herd-level identifier (specifically herd ID) to allow linkage with other key national databases.

## Supplementary Information

**Additional file 1: Figure S1. **Distribution of monthly mean SCC values among supplying farms during 2019, by calendar month. As outlined in Trader Notice DH/TN/01/2018 (revised May 2018) from DAFM to milk purchasers, this is undertaken by calculating the geometric mean based on all SCC values available within the window of interest. The percentage of supplying herds with monthly mean SCC values below 200,000 cells/mL is indicated. **Figure S2.** Distribution of monthly mean SCC values among supplying farms during 2020, by calendar month. As outlined in Trader Notice DH/TN/01/2018 (revised May 2018) from DAFM to milk purchasers, this is undertaken by calculating the geometric mean based on all SCC values available within the window of interest. The percentage of supplying herds with monthly mean SCC values below 200,000 cells/mL is indicated. **Figure S3.** Distribution of monthly mean SCC values among supplying farms during 2019, by milk purchaser. As outlined in Trader Notice DH/TN/01/2018 (revised May 2018) from DAFM to milk purchasers, this is undertaken by calculating the geometric mean based on all SCC values available within the window of interest. The percentage of supplying herds with monthly mean SCC values below 200,000 cells/mL is indicated. **Figure S4.** Distribution of monthly mean SCC values among supplying farms during 2020, by milk purchaser. As outlined in Trader Notice DH/TN/01/2018 (revised May 2018) from DAFM to milk purchasers, this is undertaken by calculating the geometric mean based on all SCC values available within the window of interest. The percentage of supplying herds with monthly mean SCC values below 200,000 cells/mL is indicated. **Figure S5.** Distribution of EMA classification (B [*'Restrict'*], C [*'Caution'*] or D [*'Prudence'*]) of in-lactation tubes sold by different milk purchasers during 2019. The percentage of in-lactation tubes with an EMA classification of C is indicated. **Figure S6.** Distribution of EMA classification (B [*'Restrict'*], C [*'Caution'*] or D [*'Prudence'*]) of in-lactation tubes sold by different milk purchasers during 2020. The percentage of in-lactation tubes with an EMA classification of C is indicated. **Figure S7.** Distribution of EMA classification (B [*'Restrict'*], C [*'Caution'*] or D [*'Prudence'*]) of dry cow tubes sold by different milk purchasers during 2019. The percentage of dry cow tubes with an EMA classification of C is indicated. **Figure S8.** Distribution of EMA classification (B [*'Restrict'*], C [*'Caution'*] or D [*'Prudence'*]) of dry cow tubes sold by different milk purchasers during 2020. The percentage of dry cow tubes with an EMA classification of C is indicated.

## Data Availability

The datasets used in the current study are not publicly available.
