# Supplementary material for: Prescribing and sales of intramammary antimicrobials in Ireland in 2019 and 2020: the role of milk purchasers
Source: Ir Vet J. 2022 Nov 19;75:20. doi: 10.1186/s13620-022-00227-4 (PMC9675076; doi:10.1186/s13620-022-00227-4)
Supplement: Supplementary file 1 — Additional file 1: Figure S1. Distribution of monthly mean SCC values among supplying farms during 2019, by calendar month. As outlined in Trader Notice DH/TN/01/2018 (revised May 2018) from DAFM to milk purchasers, this is undertaken by calculating the geometric mean based on all SCC values available within the window of interest. The percentage of supplying herds with monthly mean SCC values below 200,000 cells/mL is indicated. Figure S2. Distribution of monthly mean SCC values among supplying farms during 2020, by calendar month. As outlined in Trader Notice DH/TN/01/2018 (revised May 2018) from DAFM to milk purchasers, this is undertaken by calculating the geometric mean based on all SCC values available within the window of interest. The percentage of supplying herds with monthly mean SCC values below 200,000 cells/mL is indicated. Figure S3. Distribution of monthly mean SCC values among supplying farms during 2019, by milk purchaser. As outlined in Trader Notice DH/TN/01/2018 (revised May 2018) from DAFM to milk purchasers, this is undertaken by calculating the geometric mean based on all SCC values available within the window of interest. The percentage of supplying herds with monthly mean SCC values below 200,000 cells/mL is indicated. Figure S4. Distribution of monthly mean SCC values among supplying farms during 2020, by milk purchaser. As outlined in Trader Notice DH/TN/01/2018 (revised May 2018) from DAFM to milk purchasers, this is undertaken by calculating the geometric mean based on all SCC values available within the window of interest. The percentage of supplying herds with monthly mean SCC values below 200,000 cells/mL is indicated. Figure S5. Distribution of EMA classification (B ['Restrict'], C ['Caution'] or D ['Prudence']) of in-lactation tubes sold by different milk purchasers during 2019. The percentage of in-lactation tubes with an EMA classification of C is indicated. Figure S6. Distribution of EMA classification (B ['Restrict'], C ['Cautio [file 13620_2022_227_MOESM1_ESM.docx]

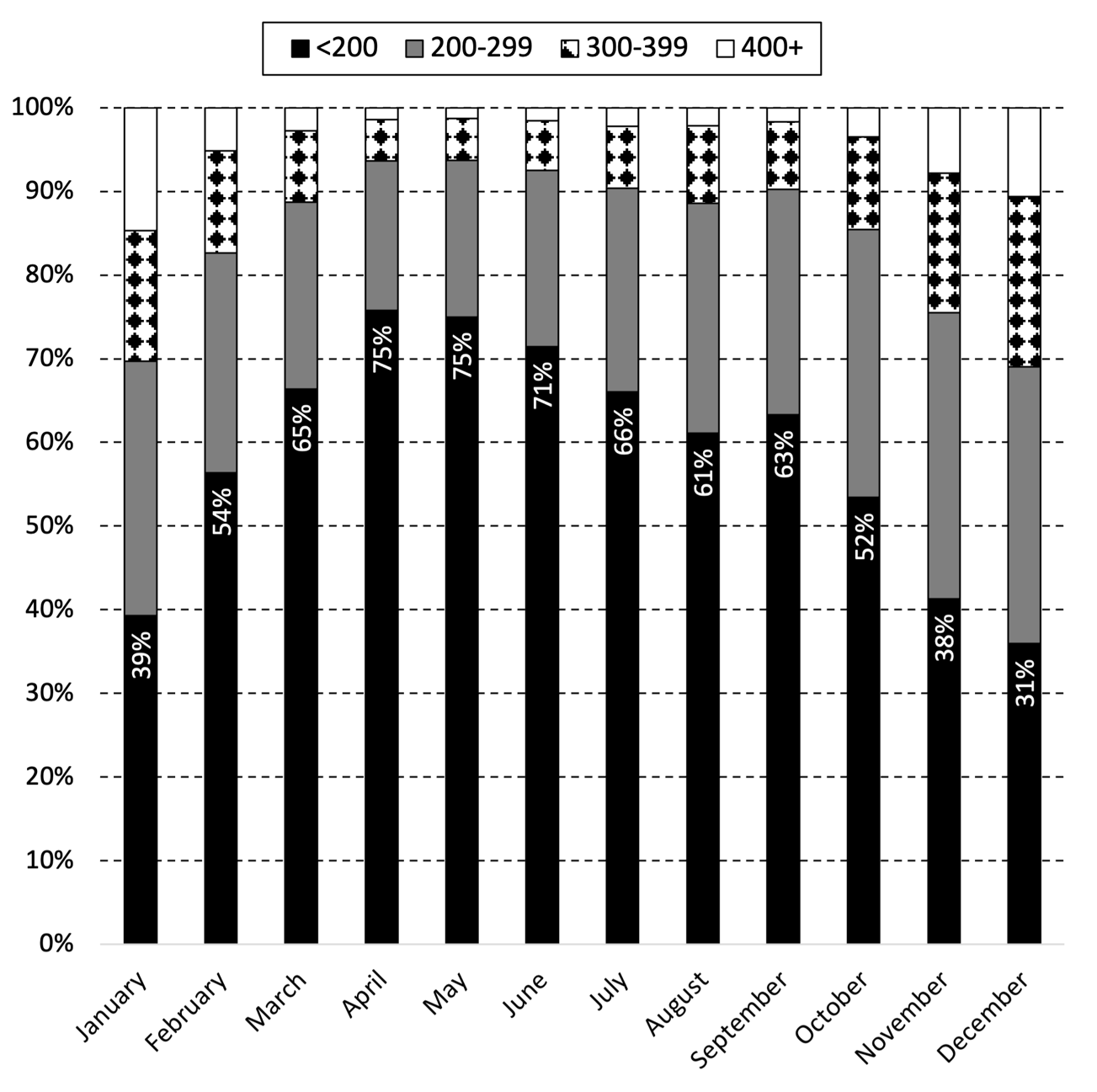


Figure S1. Distribution of monthly mean SCC values among supplying farms during 2019, by calendar month. As outlined in Trader Notice DH/TN/01/2018 (revised May 2018) from DAFM to milk purchasers, this is undertaken by calculating the geometric mean based on all SCC values available within the window of interest. The percentage of supplying herds with monthly mean SCC values below 200,000 cells/mL is indicated.


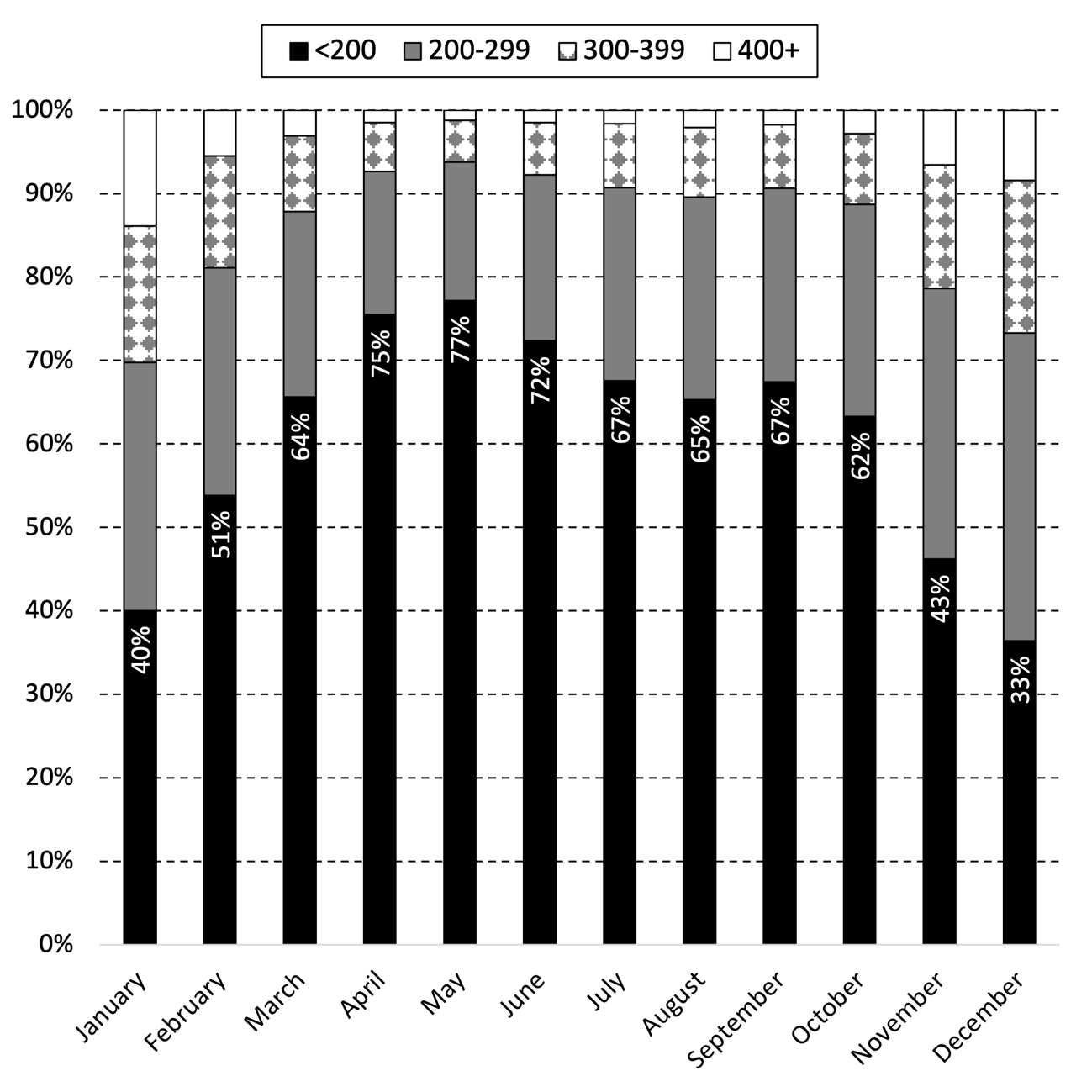


Figure S2. Distribution of monthly mean SCC values among supplying farms during 2020, by calendar month. As outlined in Trader Notice DH/TN/01/2018 (revised May 2018) from DAFM to milk purchasers, this is undertaken by calculating the geometric mean based on all SCC values available within the window of interest. The percentage of supplying herds with monthly mean SCC values below 200,000 cells/mL is indicated.


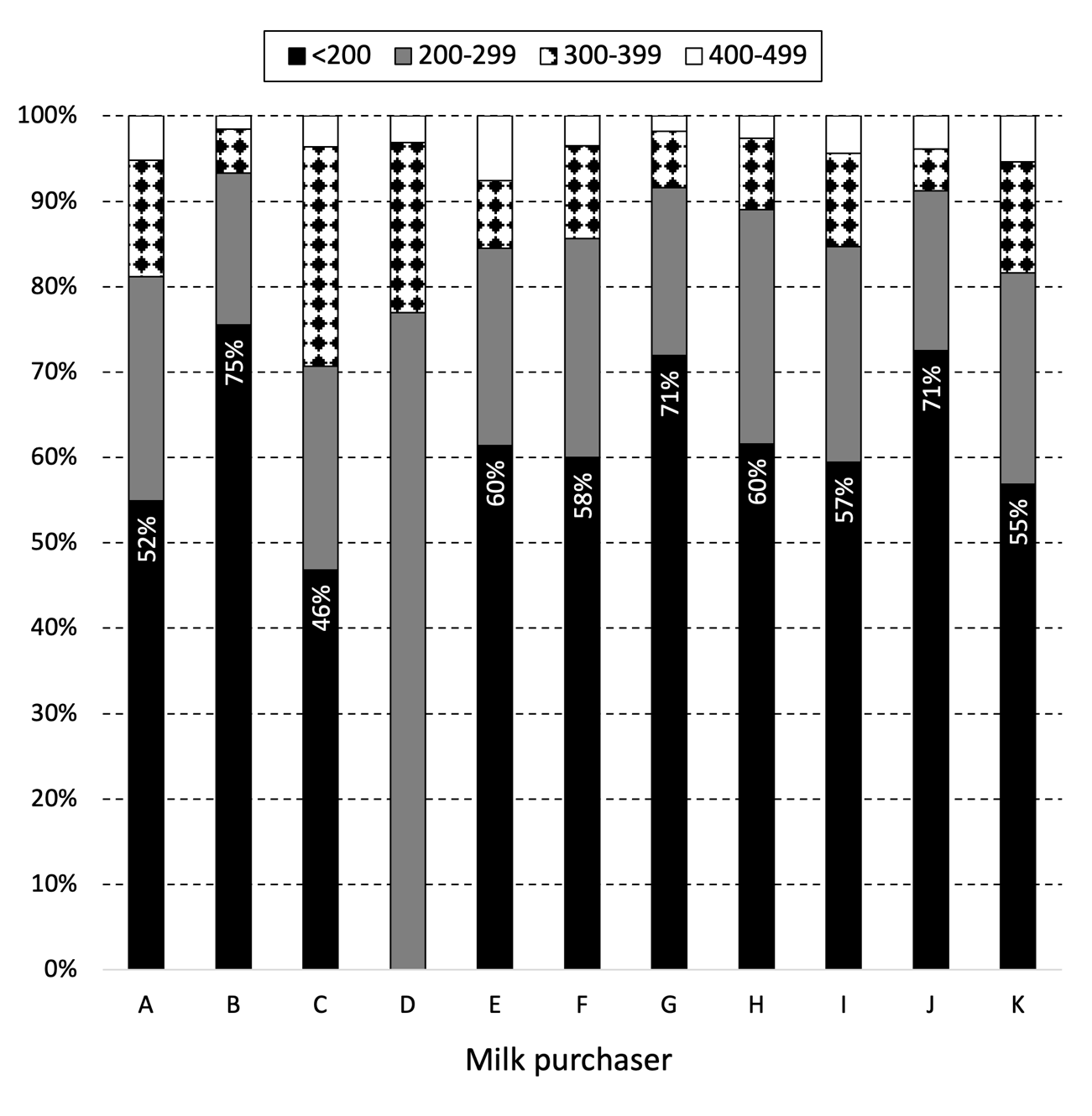


Figure S3. Distribution of monthly mean SCC values among supplying farms during 2019, by milk purchaser. As outlined in Trader Notice DH/TN/01/2018 (revised May 2018) from DAFM to milk purchasers, this is undertaken by calculating the geometric mean based on all SCC values available within the window of interest. The percentage of supplying herds with monthly mean SCC values below 200,000 cells/mL is indicated.


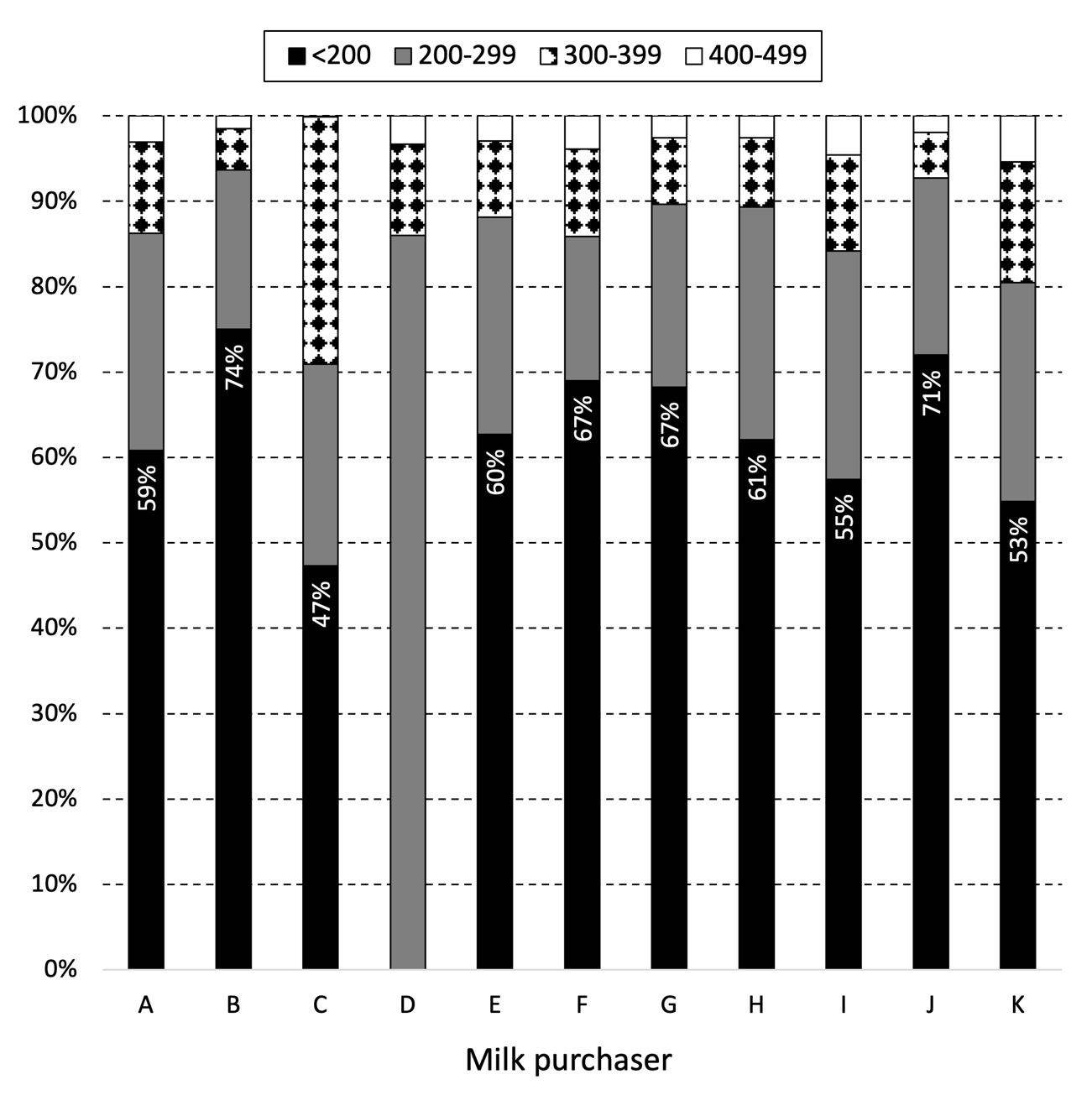


Figure S4. Distribution of monthly mean SCC values among supplying farms during 2020, by milk purchaser. As outlined in Trader Notice DH/TN/01/2018 (revised May 2018) from DAFM to milk purchasers, this is undertaken by calculating the geometric mean based on all SCC values available within the window of interest. The percentage of supplying herds with monthly mean SCC values below 200,000 cells/mL is indicated.


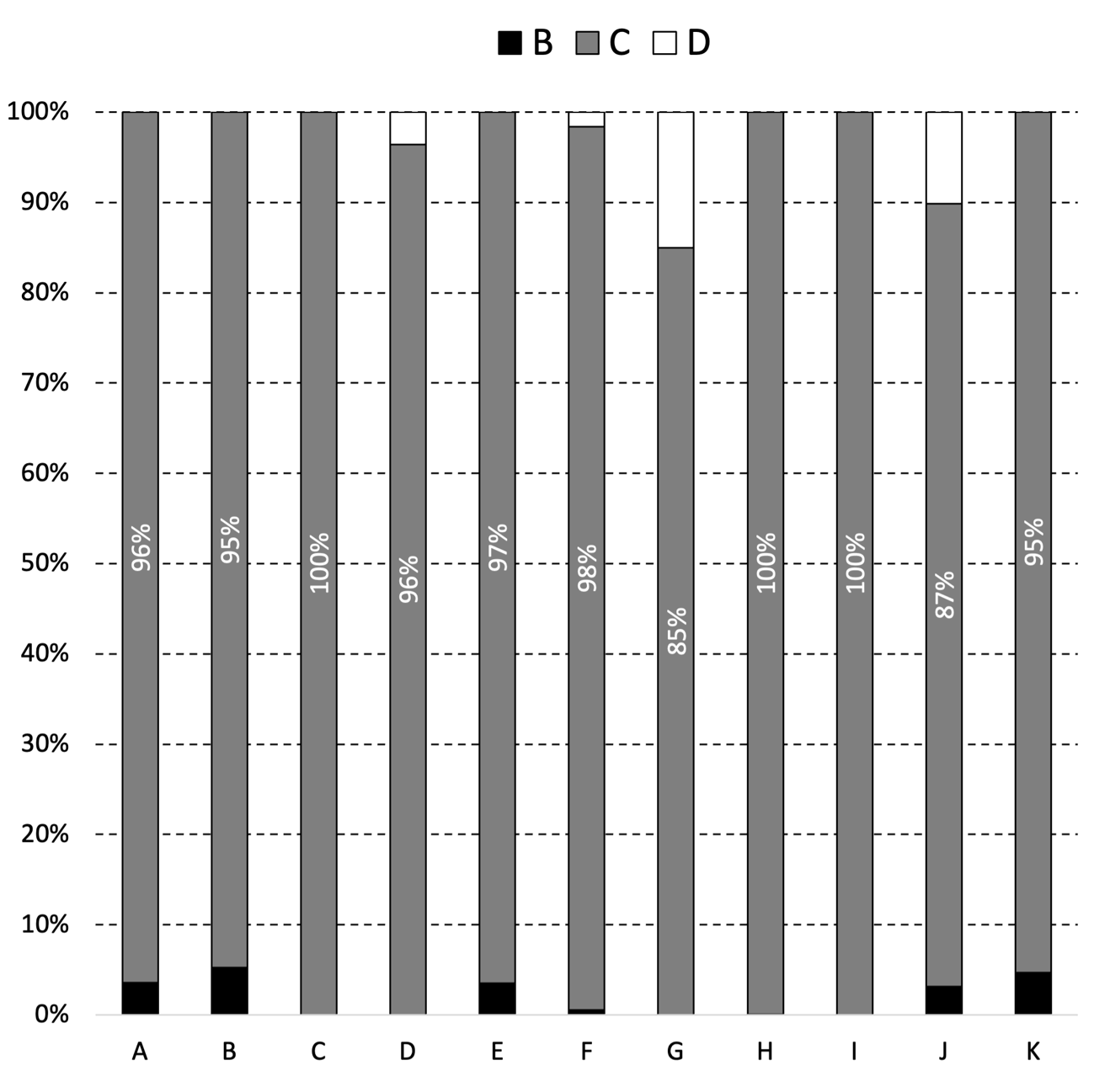


Figure S5. Distribution of EMA classification (B [*'Restrict'*], C [*'Caution'*] or D [*'Prudence'*]) of in-lactation tubes sold by different milk purchasers during 2019. The percentage of in-lactation tubes with an EMA classification of C is indicated.


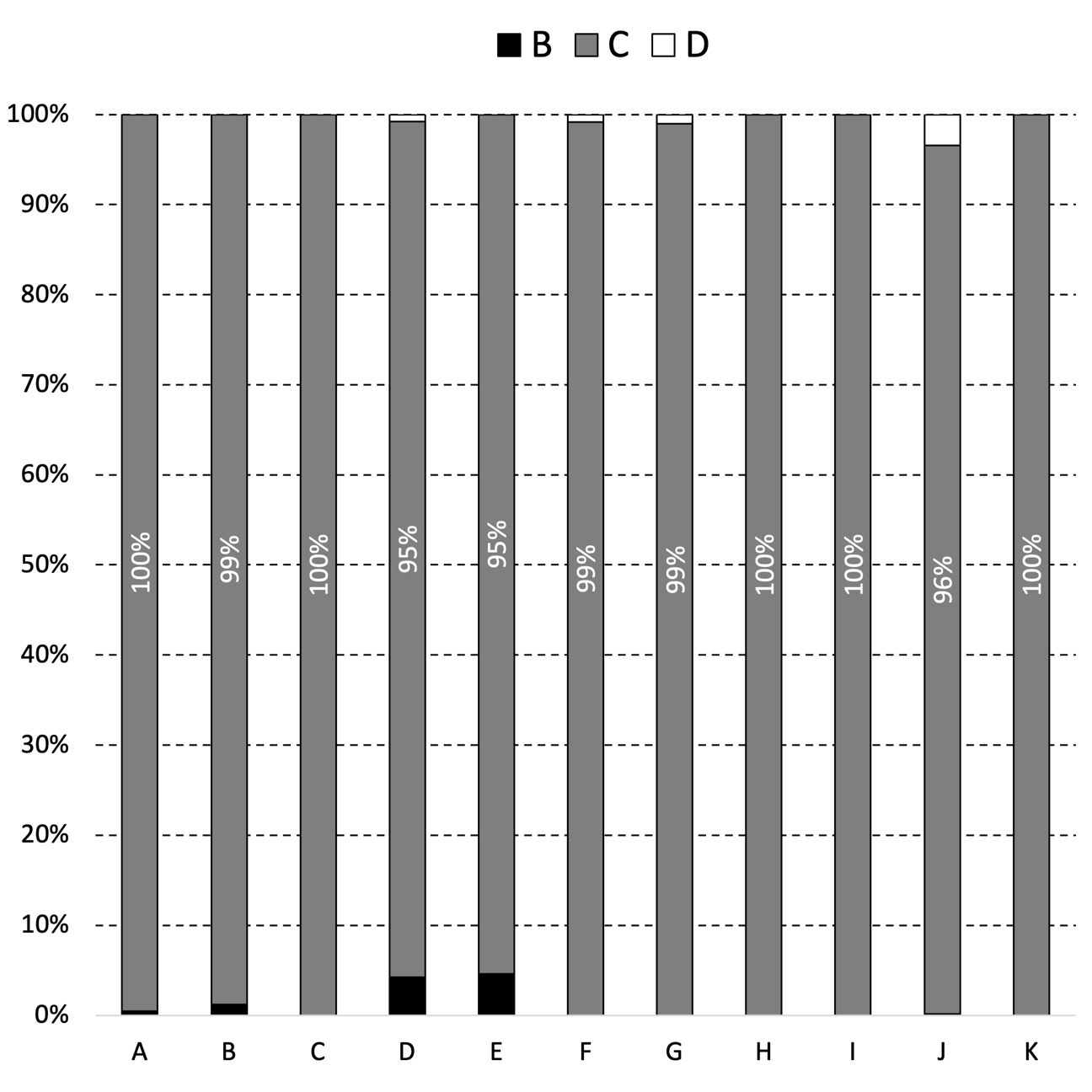


Figure S6. Distribution of EMA classification (B [*'Restrict'*], C [*'Caution'*] or D [*'Prudence'*]) of in-lactation tubes sold by different milk purchasers during 2020. The percentage of in-lactation tubes with an EMA classification of C is indicated.


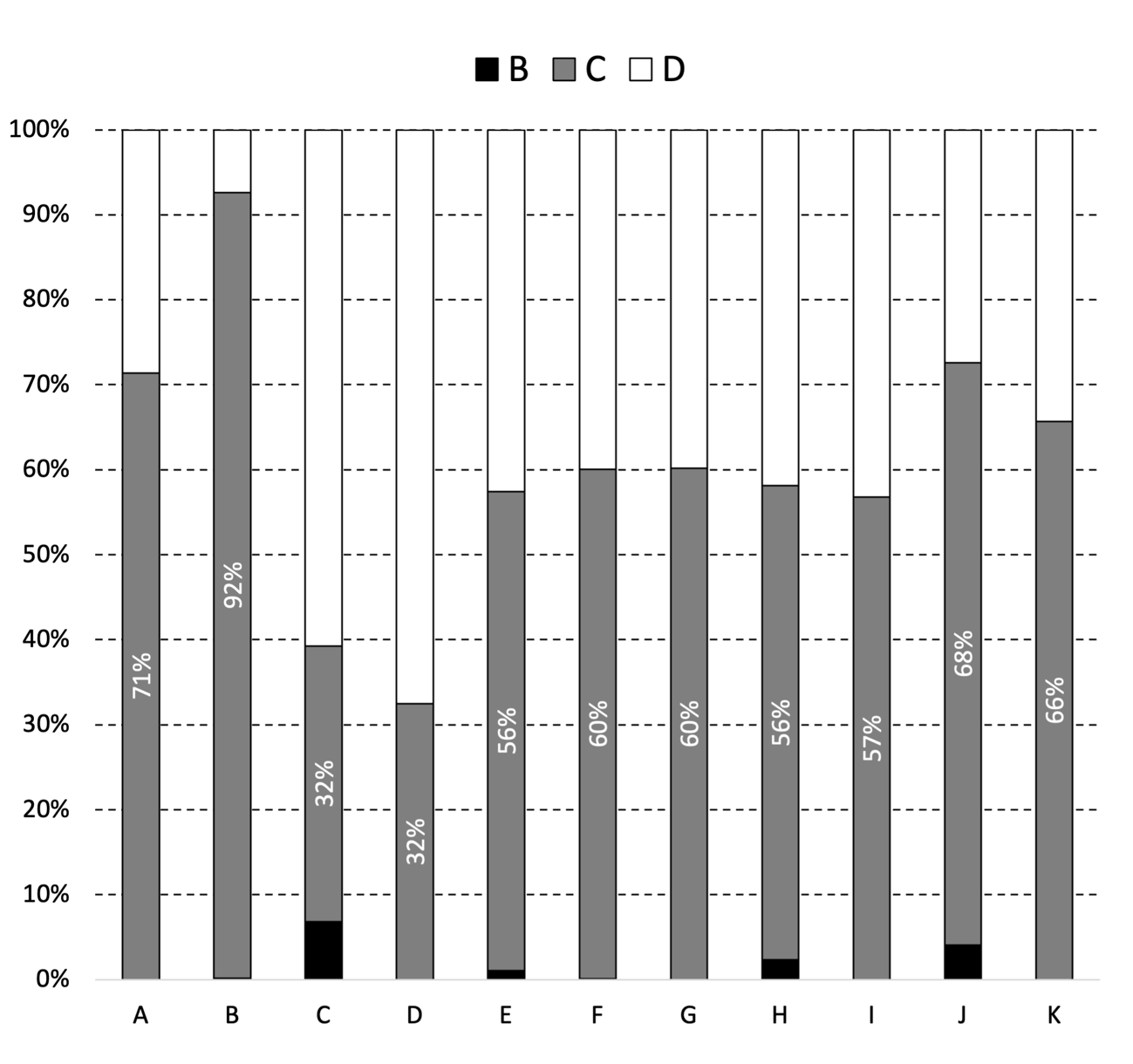


Figure S7. Distribution of EMA classification (B [*'Restrict'*], C [*'Caution'*] or D [*'Prudence'*]) of dry cow tubes sold by different milk purchasers during 2019. The percentage of dry cow tubes with an EMA classification of C is indicated.


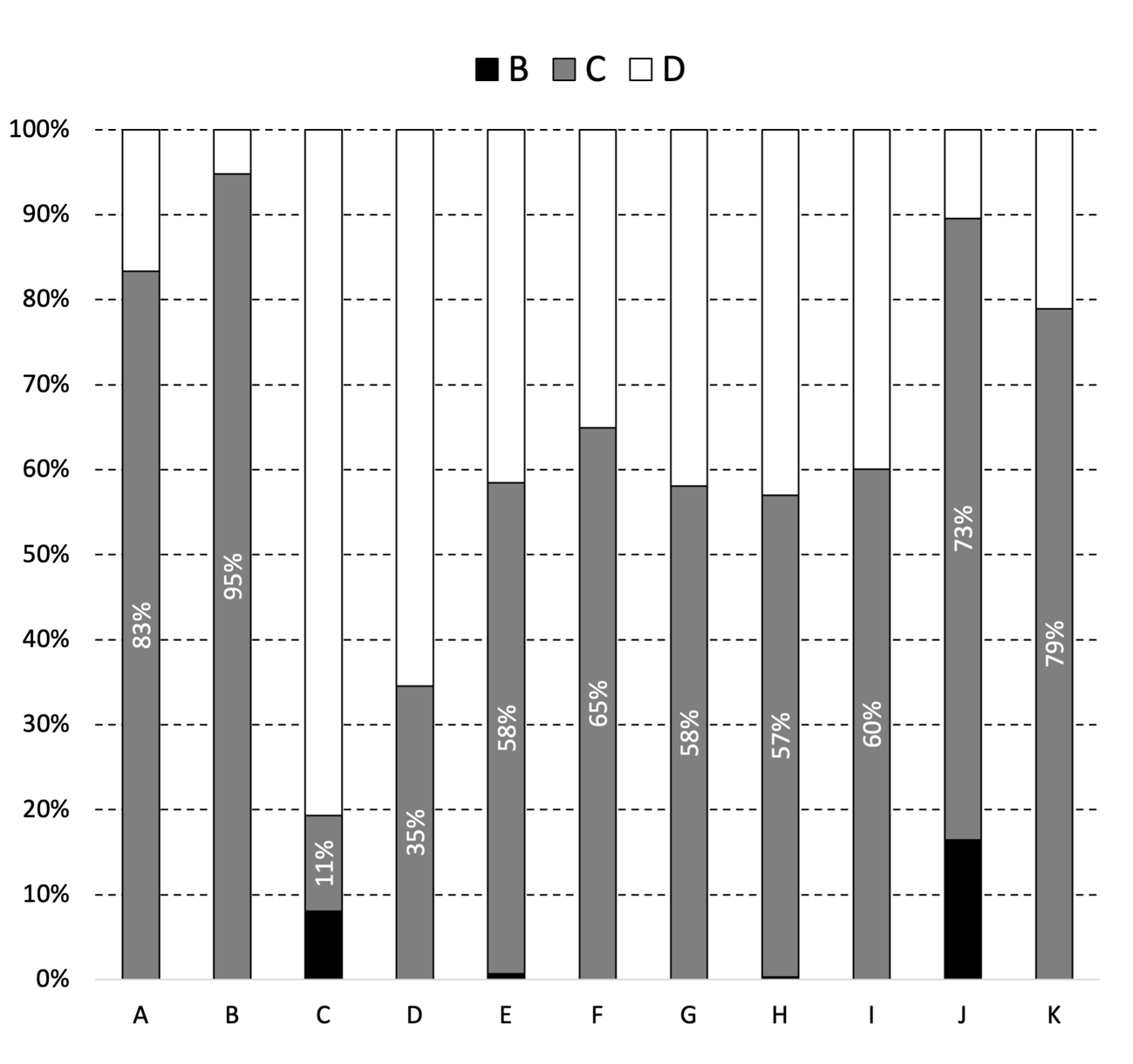


Figure S8. Distribution of EMA classification (B [*'Restrict'*], C [*'Caution'*] or D [*'Prudence'*]) of dry cow tubes sold by different milk purchasers during 2020. The percentage of dry cow tubes with an EMA classification of C is indicated.
